# An Integrative ATAC-Seq and RNA-Seq Analysis of Spleen Tissues from Largemouth Bass (*Micropterus salmoides*) Infected with Iridovirus (LMBV)

**DOI:** 10.3390/ijms27094124

**Published:** 2026-05-05

**Authors:** Hui Sun, Jixiang Hua, Yifan Tao, Siqi Lu, Wen Wang, Yalun Dong, Linbing Zhang, Jixiang He, Jie He, Jun Qiang

**Affiliations:** 1 Wuxi Fisheries College, Nanjing Agricultural University, Wuxi 214081, China; sunhhh0126@126.com (H.S.); huajixiang@foxmail.com (J.H.); linda1995king@163.com (W.W.); rspy259695@163.com (L.Z.); 2 Key Laboratory of Freshwater Fisheries and Germplasm Resources Utilization, Ministry of Agriculture and Rural Affairs, Freshwater Fisheries Research Center, Chinese Academy of Fishery Sciences, Wuxi 214081, Chinalusiqi@ffrc.cn (S.L.); dongyalun@ffrc.cn (Y.D.); 3 Fisheries Research Institute, Anhui Academy of Agricultural Sciences, Hefei 230041, China; hejixiangah@sina.com

**Keywords:** largemouth bass, LMBV, chromatin accessibility, ATAC-seq, epigenetic regulation

## Abstract

In this study, we systematically analyzed the dynamic changes in chromatin accessibility and the transcriptional responses in the spleen of largemouth bass (*Micropterus salmoides*) following infection with iridovirus (LMBV) using the assay for transposase-accessible chromatin with sequencing (ATAC-seq) and transcriptome sequencing (RNA-seq). Based on post-infection survival status, largemouth bass were classified into a resistant group (SR) and a susceptible group (SS). A total of 11,317 differentially accessible regions were identified between the two groups, among which the chromatin accessibility of core promoter regions was entirely increased in the SR group, suggesting that chromatin remodeling in these regions may directly participate in the transcriptional regulation of immune-related genes. Functional enrichment analysis revealed that genes associated with differentially accessible regions were significantly enriched in immune-related pathways such as autophagy, apoptosis, Toll-like receptor signaling, and NOD-like receptor signaling. Motif analysis further identified that transcription factors significantly enriched in the SR group included CTCF and heterodimers composed of multiple members of the ETS and FOX transcription factor families. Through integrative analysis, seven transcription factors (CTCF, Spi1, ETV2::FOXI1, FOXJ2::ELF1, FOXO1::ELK1, SPIC, and FOXO1::ELF1) were found to be significantly enriched in core promoter regions. To further screen for differentially expressed genes directly regulated by chromatin accessibility changes, an overlapping analysis was performed between 629 predicted target genes and 2656 differentially expressed genes (DEGs), resulting in the identification of 71 candidate genes. Among these, three immune-related genes (*irf4a*, *btk*, and *nfil3-2*) belonging to the ETS and FOX families were identified. This study reveals the dynamic chromatin accessibility landscape of largemouth bass in response to LMBV infection and demonstrates that increased chromatin accessibility in core promoter regions is closely associated with the resistant phenotype. Heterodimers of ETS and FOX family transcription factors may participate in antiviral immune responses by regulating the expression of key immune genes such as *irf4a*, *btk*, and *nfil3-2*, providing potential epigenetic molecular markers for disease resistance breeding in fish.

## 1. Introduction

In China’s freshwater aquaculture industry, the largemouth bass (*Micropterus salmoides*) represents a species of considerable economic significance, favored by consumers for its tender texture, unique flavor, and balanced nutritional composition [1]. Driven by its fast growth rate and strong adaptation capacity [2], largemouth bass has undergone rapid expansion in aquaculture scale since its introduction to China in the 1980s and has now become a major aquaculture species in southern China, particularly in Guangdong, Jiangsu, and Zhejiang provinces [3]. However, with the scaling up of breeding and more crowded rearing conditions, disease outbreaks in largemouth bass have become increasingly frequent, and issues related to bacterial, viral, and parasitic infections have become more prominent [4,5,6]. Among these, the disease caused by largemouth bass iridovirus (LMBV) is particularly severe [7], leading to large-scale mortality and significant economic losses during aquaculture, seriously constraining the sustainable development of the industry. LMBV infection can cause darkening of body color, gill hemorrhage, and enlargement and necrosis of internal organs [4,8], resulting in a mortality rate as high as over 60% [9,10,11,12], causing huge economic losses to farmers and severely threatening the healthy and sustainable development of the industry. Currently, the prevention and control of LMBV disease mainly rely on chemical agents such as disinfectants and antibiotics [13]; however, these measures have limitations, including unstable efficacy and drug residues. Therefore, enhancing the resistance of largemouth bass to LMBV and breeding new disease-resistant varieties are considered important approaches to achieving disease prevention and control and ensuring the sustainable development of the industry.

LMBV typically targets lymphoid and hematopoietic organs, with the spleen being the most important lymphoid organ in fish [14]. The spleen plays an irreplaceable role in the host’s immune defense, serving as the core site for antigen presentation and the initiation of specific immune responses. The spleen contains abundant melanomacrophage centers (MMCs), which play critical roles in antigen capture, immune memory, and the regulation of inflammation [15]. Furthermore, the spleen is a key site for B cell activation, antibody production, and T cell proliferation and differentiation, all of which are essential for initiating both specific and non-specific immune responses. Following iridovirus infection in largemouth bass, the spleen, as the primary target organ for viral replication, exhibits pronounced pathological changes, including splenomegaly, activation of MMCs, lymphocyte infiltration, and tissue necrosis [16]. Due to its diversity of cell types and immune pathways, the spleen is commonly used to characterize host responses to pathogen stimulation. For instance, in Atlantic salmon (*Salmo salar*) infected with piscine reovirus, viral infection was found to induce the expression of antiviral response genes in the spleen [17]. In groupers (*Epinephelus coioides*) infected with Singapore grouper iridovirus (SGIV), the expression levels of CXCL8, CXCL12a, CXCR4a, and CXCR1b were significantly upregulated in the spleen of resistant individuals, indicating that the spleen plays an important role in antiviral immunity [18].

Disease resistance in fish refers to the ability to resist pathogenic microbial infection and ultimately maintain body homeostasis through a series of complex physiological, biochemical, and immunological mechanisms under natural or artificial aquaculture conditions. As the second most important trait in aquaculture after growth performance, disease resistance directly determines the survival rate and yield of aquatic animals [19,20]. Genetically, disease resistance in fish typically exhibits low to moderate heritability, with estimates ranging approximately from 0.05 to 0.35 [21,22,23], indicating that genetic improvement for this trait can be achieved through selective breeding. Despite this potential, the genetic architecture of disease resistance is highly complex; it is not controlled by a single gene but is a typical complex quantitative trait regulated by multiple genetic loci [24,25]. Currently, methods such as genome-wide association studies (GWAS) and RNA sequencing (RNA-seq) have been widely applied to identify candidate genes associated with LMBV resistance in largemouth bass [26]. For example, GWAS analysis identified ten candidate genes related to LMBV resistance, nine of which were enriched in the FoxO signaling pathway [20]. Our team previously found that a selectively bred population of largemouth bass exhibited a significant 28.9% increase in 7-day survival rate, and through whole-genome resequencing, we identified genes such as *atp7b*, *nampt*, *hpcal4*, and *ppsst1/2* [27]. Additionally, RNA-seq analysis of the spleen from diseased largemouth bass detected differential expression of 6254 genes, with functional annotation revealing enrichment in immune pathways such as endocytosis, the JAK-STAT signaling pathway, and the MAPK signaling pathway [28]. These studies have revealed the genetic basis of LMBV resistance in largemouth bass at both the DNA sequence and transcriptional levels. However, in addition to DNA sequence variation, epigenetic modifications, as regulatory mechanisms that do not alter the sequence, also play crucial roles in gene expression regulation.

Epigenetics refers to heritable regulation of gene expression achieved through mechanisms such as DNA methylation, histone modification, chromatin remodeling, and non-coding RNAs [29]. Among these, chromatin accessibility directly reflects whether a given region is in an “open” or “closed” functional state [30]. Open chromatin regions carry over 90% of the binding sites for transcription factors (TFs) [31] and can interact with TFs to regulate growth, development, and cell differentiation processes [32]. The development of the assay for transposase-accessible chromatin with high-throughput sequencing (ATAC-seq) has provided a tool for sensitively mapping open chromatin regions across the genome. This technology utilizes hyperactive Tn5 transposase to specifically cleave open chromatin regions, allowing precise localization of nucleosome positions and accessible chromatin areas [33,34]. In recent years, chromatin accessibility has been increasingly applied to study immune responses in various fish species, revealing that pathogen infection is often accompanied by significant changes in the chromatin accessibility of host immune regulatory elements, which are associated with enhanced expression of relevant genes [35]. For instance, in a study on the head kidney of turbot (*Scophthalmus maximus*) infected with poly I:C virus, combined ATAC-seq and RNA-seq analysis revealed widespread changes in chromatin status following immune stimulation in both control and infected groups, which were closely correlated with changes in differentially expressed genes (DEGs) [36]. Similarly, in the head kidney of large yellow croaker (*Larimichthys crocea*) infected with LMBV, changes in chromatin accessibility in regulatory regions of key immune-related genes and altered expression of the AP-1 transcription factor (including the *Batf* gene) were observed [37]. Similar phenomena have also been validated in Nile tilapia (*Oreochromis niloticus*) infected with *Streptococcus agalactiae* [38]. Collectively, these studies indicate that chromatin accessibility, as a key epigenetic regulatory mechanism, is a crucial influencing factor in the immune response of aquatic animals. However, systematic research on the regulatory mechanism of chromatin accessibility in the spleen of largemouth bass following LMBV infection is currently lacking.

This study systematically analyzed the chromatin accessibility landscape of largemouth bass spleen tissue following LMBV infection using ATAC-seq technology. By further integrating RNA-seq data, we identified key transcription factors of the ETS and FOX families as well as genes associated with disease resistance. From an epigenetic perspective, this study reveals the immune regulatory mechanism underlying LMBV resistance in largemouth bass, providing theoretical support for molecular breeding of disease-resistant varieties.

## 2. Results

### 2.1. Phenotypic Variation in the Spleen of Largemouth Bass Infected with LMBV

**Figure 1:**
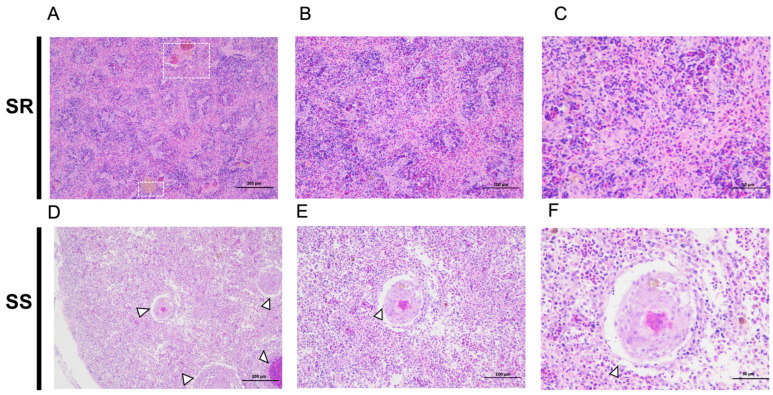
HE staining of largemouth bass spleen infected with LMBV. (**A**–**C**) SR group infected with LMBV. Melanomacrophages are indicated by white squares. (**D**–**F**) SS group infected with LMBV. Typical granulomatous structures in the spleen tissue are indicated by white triangles. The white dashed boxes indicate representative pathological changes in the SR group, and arrowheads point to granulomatous lesions in the SS group. Scale bars: 200 μm (**A**,**D**); 100 μm (**B**,**E**); 50 μm (**C**,**F**).

Histopathological examination revealed that the degree of spleen tissue damage in the SS and SR groups was highly correlated with disease resistance. In the SR group, the morphology of splenic cells was largely normal, with clear structures of lymphocytes and macrophages, and only a small number of cells showed degeneration or necrosis. No obvious interstitial edema or hemorrhage was observed (Figure 1A–C). In contrast, the SS group exhibited significant macrophage infiltration and pronounced subcapsular necrosis in the spleen tissue (Figure 1D–F). Moreover, typical granulomatous structures were observed in the spleen tissue of the SS group, characterized by central necrosis appearing dark purple, surrounded by proliferating epithelioid cells.

### 2.2. Chromatin Accessibility in the Spleen of Largemouth Bass

**Figure 2:**
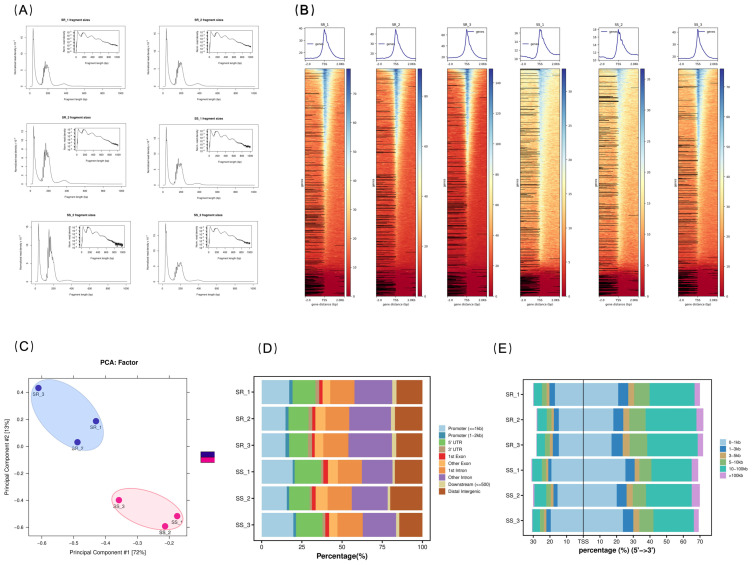
Examination of ATAC-seq data quality. (**A**) Fragment length distribution in the SS and SR groups. X-axis: fragment length (bp); Y-axis: percentage of total fragments. (**B**) Upper panel: average read depth of ATAC-seq signals around the transcription start site (TSS); lower panel: enrichment of ATAC-seq signals around the TSS. (**C**) Principal component analysis (PCA) plot. (**D**) Distribution of common peaks between the SS and SR groups. Genomic functional regions include promoters, 5′ UTR, 3′ UTR, introns, exons, downstream regions, and distal intergenic regions. (**E**) Distribution of TFs across the TSS-flanking regions.

To evaluate the quality of the ATAC-seq data, we examined the library fragment length distribution, read length distribution, alignment rate to the reference genome, and enrichment around transcription start sites (TSS). After removing low-quality reads, a total of 747,176,206 clean reads were obtained, and following stringent quality filtering, 743,913,262 high-quality (HQ) clean reads remained. Of these, over 96.33% of the clean reads were successfully mapped to the largemouth bass reference genome. Detailed information on the data is presented in Table S1. An expected distribution of fragment lengths was observed across all libraries (Figure 2A). The fragment length distribution revealed a prominent peak at approximately 40 bp, indicative of Tn5 transposase insertion events occurring in nucleosome-depleted regions. A second peak appeared just below 200 bp, representing insertions flanking mononucleosomes. A total of 30,027 peaks were identified in the SS group, and 12,077 peaks were identified in the SR group. ATAC-seq signals were largely concentrated near TSS (Figure 2B). Principal component analysis (PCA) (Figure 2C) showed that the first principal component (PC1) explained 72% of the total variance, clearly separating the susceptible (SS) and resistant (SR) groups. The clear clustering of the SS and SR groups along the PC1 axis indicates systematic differences in chromatin accessibility between the two groups. Furthermore, biological replicates within each group clustered well, demonstrating high data reproducibility. Functional annotation of all peaks revealed that the majority of peaks were located in promoter regions and introns (Figure 2D). The peaks with the most transcription factor binding sites were located 0–1 kb and 10–100 kb from the TSS (Figure 2E). These data indicate that chromatin accessibility in core promoter regions plays a crucial role in the transcriptional regulation of genes.

### 2.3. Analysis of Differentially Accessible Chromatin Regions (DARs) Between Groups

**Figure 3:**
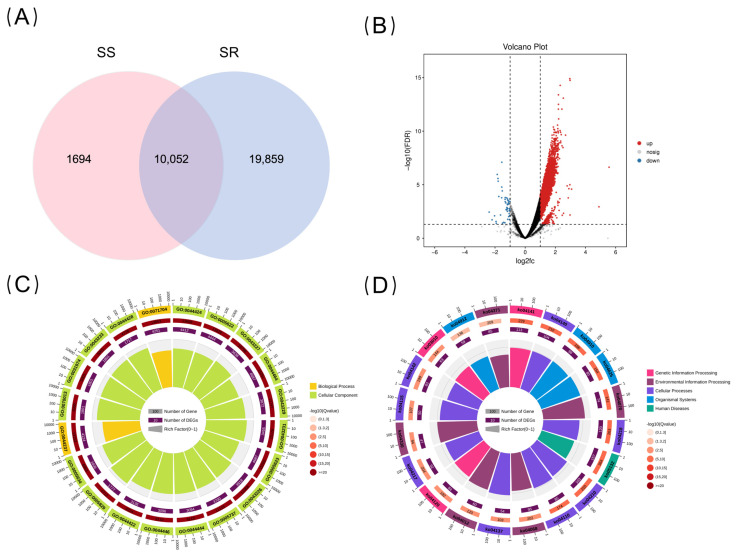
Identification and functional annotation of genes corresponding to differential peaks. (**A**) Venn diagram of genes corresponding to differential peaks between the two groups. (**B**) Volcano plot comparing genes corresponding to differential peaks, with red and blue indicating differential genes corresponding to differential peaks with increased and decreased accessibility, respectively. (**C**) GO functional classification circle plot of genes corresponding to differential peaks. (**D**) KEGG pathway classification circle plot of genes corresponding to differential peaks.

To characterize the composition of open chromatin regions between the SR and SS groups, we first analyzed the peaks identified in both groups. The results showed a total of 31,605 peaks (Table S2), including 1694 peaks unique to the SR group, 19,859 peaks unique to the SS group, and 10,052 peaks shared between the two groups (Figure 3A). Based on this, we performed differential accessibility analysis using DiffBind (version 2.8.0). With a threshold of log_2_ fold change ≥ 1 and FDR ≤ 0.05, we identified 11,317 (Table S3) chromatin regions with statistically significant differences between the two groups (differentially accessible regions, DARs). Compared to the SS group, 11,267 regions exhibited increased accessibility in the SR group, while 50 regions showed decreased accessibility (Figure 3B). In terms of genomic distribution, DARs observed in promoter regions accounted for 14.6% (2893 DARs). Notably, among the DARs located in core promoter regions (within 0.5 kb upstream of the transcription start site), the vast majority (over 99%) exhibited increased accessibility, indicating that core promoter regions play an important role in transcriptional regulation. The top 10 DARs with the most significantly increased accessibility are listed in Table S4.

To explore the potential functions of the genes associated with these differential peaks, GO (Figure 3C) and KEGG (Figure 3D) enrichment analyses were performed. GO analysis revealed that the biological processes (BP) were mainly related to cellular metabolic process, organic substance metabolic process, and primary metabolic process. Cellular component (CC) functions were primarily enriched in the intracellular part and intracellular and membrane-bounded organelles. Molecular functions (MFs) were mainly enriched in enzyme binding, organic cyclic compound binding, and heterocyclic compound binding. Furthermore, KEGG enrichment analysis showed that the genes corresponding to differential peaks were significantly enriched in three categories of immune-related pathways: (1) cell-clearance-related pathways (Autophagy, Apoptosis, Necroptosis, Lysosome, and Endocytosis), with 532 genes enriched; (2) stress and cell cycle regulation-related pathways (p53, FoxO, Insulin signaling, and Cell cycle), with 328 genes enriched; and (3) pathogen recognition-related pathways (Toll-like receptor, NOD-like receptor, and C-type lectin receptor), with 218 genes enriched. These results indicate that DAR-associated genes are widely involved in both innate and adaptive immune regulation and that changes in chromatin accessibility may represent an important epigenetic mechanism underlying enhanced disease resistance.

### 2.4. Transcriptome Data Analysis

**Figure 4:**
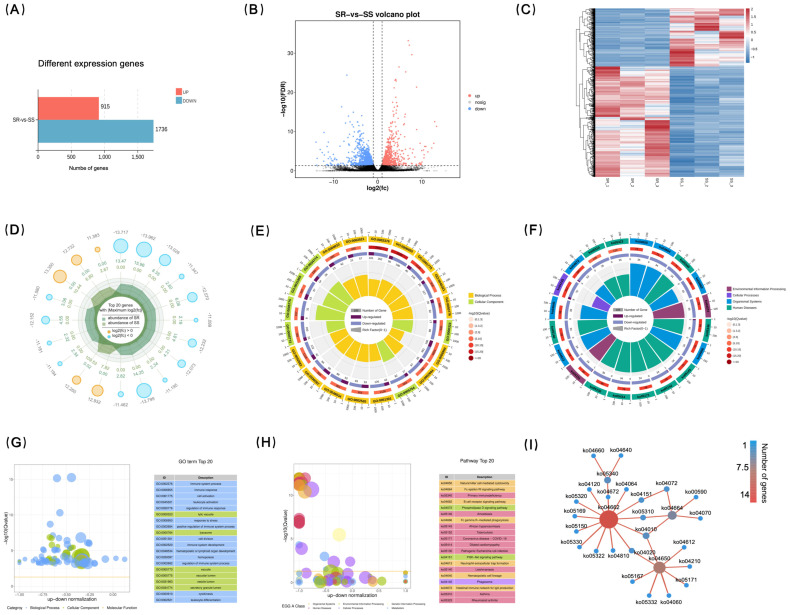
Transcriptome profiling of spleen from SR and SS groups following iridovirus infection. (**A**) Bar chart of DEGs. (**B**) Volcano map of DEGs. (**C**) Clustering heatmap of differentially expressed genes (DEGs). Color scale reflects correlation strength, with red representing positive correlation and blue negative correlation. (**D**) Radar plot of the top 20 DEGs. (**E**) Circle plot of GO functional classification. (**F**) Circle plot of KEGG pathway classification. (**G**) Bubble plot of GO enrichment analysis showing the top 20 enriched terms. (**H**) Bubble plot of KEGG pathway enrichment analysis showing the top 20 enriched pathways. (**I**) Network diagram of KEGG core genes; node size represents the number of associated genes.

To elucidate the transcriptional response of largemouth bass to LMBV infection, spleen tissues were collected after treatment for RNA sequencing. A total of 246,633,522 raw reads were obtained. After adapter trimming and removal of low-quality reads, 245,902,786 clean reads were retained, with a mapping rate exceeding 94.80% (Table S5). A total of 2651 differentially expressed genes (DEGs) were identified between the SR and SS groups (Figure 4A) (Table S6), including 915 up-regulated genes and 1736 down-regulated genes. A volcano plot visually displays the distribution of DEGs between the two groups (Figure 4B). The overall trend showed that most DEGs were highly expressed in the SR group and lowly expressed in the SS group, indicating significant differences in gene expression profiles between the groups (Figure 4C). A radar plot of the top 20 DEGs based on |log_2_FC| is shown in Figure 4D.

GO and KEGG enrichment analyses were performed on all differentially expressed genes. The top 20 enriched GO terms were mainly concentrated in the Biological Process (BP) and Cellular Component (CC) categories (Figure 4E,G). Among BP, the most significantly enriched terms included immune system process, immune response, and cell activation. In the CC category, the most significantly enriched term was lytic vacuole. KEGG pathway analysis (Figure 4F,H) revealed that the most significantly enriched pathways included natural-killer-cell-mediated cytotoxicity (35 genes), Fc epsilon RI signaling pathway (34 genes), Primary immunodeficiency (34 genes), and B cell receptor signaling pathway (40 genes). The pathways with the largest numbers of enriched genes were Phagosome (79 genes), Intestinal immune network for IgA production (56 genes), and Protein processing in endoplasmic reticulum (53 genes). Based on the KEGG enrichment results, a pathway interaction network was constructed (Figure 4I). The results showed that the B cell receptor signaling pathway, which contained the largest number of genes (14 genes), clustered with several other immune-related pathways to form a tight functional module, suggesting their synergistic roles in the antiviral immune response.

**Figure 5:**
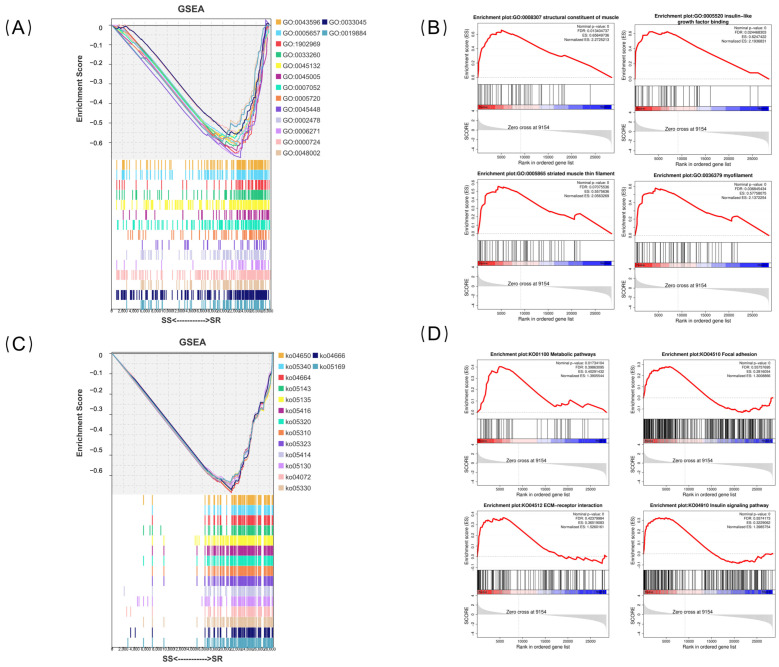
GSEA analysis of spleen tissue from SR and SS groups. (**A**) Overview of GSEA-GO enrichment. (**B**) Enrichment score (ES) curves of the top 4 pathways with the highest NES values in GSEA-GO analysis. (**C**) Overview of GSEA-KEGG enrichment. (**D**) Enrichment score (ES) curves of the top 4 pathways with the highest NES values in GSEA-KEGG analysis. The red line in the figure shows the dynamic enrichment trend of the gene set across the ranked list of differentially expressed genes.

To investigate the molecular mechanisms underlying the resistance of largemouth bass to iridovirus infection, GSEA was performed on the DEGs (Figure 5A). The GSEA-GO analysis revealed that, compared to the SR group, multiple biological pathways were significantly down-regulated in the SS group (FDR < 0.05). The top four most significantly down-regulated pathways were all related to DNA replication and mitosis (Figure 5B), including nuclear replication fork (NES = −1.996), replication fork (NES = −1.996), mitotic DNA replication (NES = −1.988), and nuclear DNA replication (NES = −1.973). Furthermore, several pathways associated with immune responses were also significantly down-regulated in the SS group, including the antigen processing and presentation of exogenous peptide antigen (NES = −1.914), the T cell receptor signaling pathway (NES = −1.667), and the innate immune response activating cell surface receptor signaling pathway (NES = −1.714). These results indicate that the SS group exhibited significant inhibition of cell cycle progression and DNA replication capacity, as well as impaired immune functions such as antigen presentation and lymphocyte activation.

The GSEA-KEGG analysis revealed that, compared to the SR group, multiple biological pathways were significantly down-regulated in the SS group (FDR = 0.05) (Figure 5C). The top four most significantly down-regulated pathways were all related to immune responses (Figure 5D), including Natural-killer-cell-mediated cytotoxicity (NES = −2.133), Primary immunodeficiency (NES = −2.117), Fc epsilon RI signaling pathway (NES = −2.112), and African trypanosomiasis (NES = −2.103). These results indicate that in the SS group, NK cell-mediated cytotoxicity, B cell development, and mast cell-mediated allergic responses were significantly suppressed. Furthermore, several other immune-related pathways were also significantly down-regulated in the SS group, including the B cell receptor signaling pathway (NES = −2.032), NF-kappa B signaling pathway (NES = −2.047, FDR = 0.000), Intestinal immune network for IgA production (NES = −2.061), and the T cell receptor signaling pathway (NES = −1.802). These findings suggest that both innate and adaptive immune functions are broadly suppressed in the SS group, with the B cell-mediated humoral immune response particularly appearing to be in a relatively quiescent state.

### 2.5. Integrated Analysis and Validation of ATAC-Seq and RNA-Seq Data

To investigate the association between changes in chromatin accessibility and transcriptional expression, we performed genomic annotation of 11,317 DARs and obtained 10,072 DAR-related genes. Subsequently, we assessed whether these DAR-related genes were significantly enriched among the DEGs identified by RNA-seq. The results showed that 687 genes overlapped between the DAR-related genes and the 2651 DEGs. Further analysis of the directional consistency between chromatin accessibility changes and gene expression changes in these overlapping genes revealed that 502 genes (73.1%) exhibited consistent alterations, i.e., increased chromatin accessibility accompanied by upregulated gene expression. These findings provide strong evidence for a high degree of synergy between chromatin remodeling and transcriptional reprogramming.

TF motif analysis was performed on the differential peaks between groups. The analysis showed that the numbers of TF motifs that were enriched in a statistically significant fashion in the SS and SR groups were 2 and 54, respectively. The most significantly changed motifs in the SS group were Spi1 and IKZF1. The top five motifs in the SR group were CTCF, ETV2::FOXI1, ELF2, Erg, and ERF::FOXI1, four of which belong to the ETS and FOX families of transcription factors.

**Figure 6:**
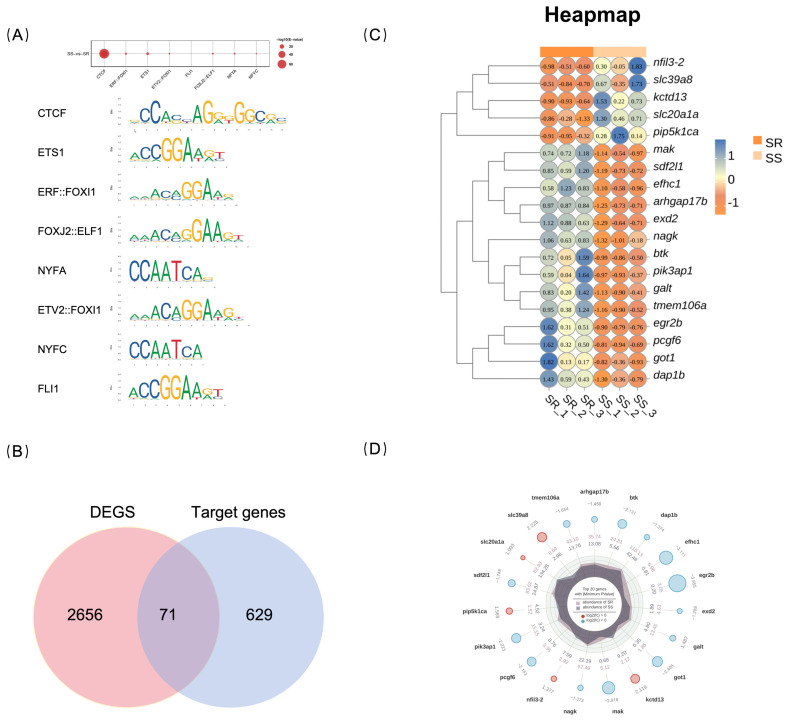
(**A**) Bubble plot of enriched differential TF motifs between the SS and SR groups. Bubble size represents the enrichment level. The motifs below correspond to the DNA sequences recognized by the transcription factors. (**B**) Venn diagram depicting the intersection between DEGs and target genes of the seven transcription factors. (**C**) Heatmap of the top 20 candidate genes based on FDR values. (**D**) Radar plot of the top 20 candidate genes based on FDR values.

Analysis of differentially enriched transcription factor (TF) binding motifs between the SR and SS groups identified a total of 132 TFs, of which 61 showed statistically significant enrichment (E-value 0.05 and FDR 0.05). Notably, all of these significantly enriched TFs were up-regulated (Table S8). The top eight most significantly enriched TFs were, in order, CTCF, ERF::FOXI1, ETS1, ETV2::FOXI1, FLI1, FOXJ2::ELF1, NFYA, and NFYC (Figure 6A). Among these, the chromatin architecture protein CTCF exhibited the most significant signal and displayed sequence diversity, being identified among the top three motifs. This suggests that CTCF may play an important role in maintaining higher-order chromatin structure in largemouth bass. Furthermore, multiple composite binding motifs involving ETS and FOX family members (e.g., ERF::FOXI1, ETV2::FOXI1) were also significantly enriched, indicating that these two families of transcription factors may participate in antiviral transcriptional regulation through cooperative binding. From the 61 TFs, seven that were significantly enriched in the core promoter region were identified as potential direct regulators: CTCF, Spi1, ETV2::FOXI1, FOXJ2::ELF1, FOXO1::ELK1, SPIC, and FOXO1::ELF1. Analysis revealed that heterodimeric composite motifs formed by ETS and FOX family members were identified among the genetic variant TFs specific to the SR and SS groups, as well as among the differential TFs between the two groups, suggesting that these two families of transcription factors may cooperate to regulate antiviral immune transcription.

The seven aforementioned TFs were collectively annotated to 629 putative target genes. Intersection of these genes with the 2651 DEGs yielded 71 candidate genes (Figure 6B), including *irf4a*, *btk*, and *nfil3-2*, among others (Table S9). These genes contain core TF binding sites within their promoter regions and are differentially expressed between the two groups; therefore, they likely represent downstream target genes directly regulated by these TFs and participate in the disease resistance response of largemouth bass. Figure 6C presents a heatmap of the top 20 highly differentially expressed genes, showing marked expression differences between the SR and SS groups. The radar chart (Figure 6D) clearly illustrates these genes’ expression profile differences between the two groups.

### 2.6. Validation of Key Transcription Factors and Gene Expression Levels

**Figure 7:**
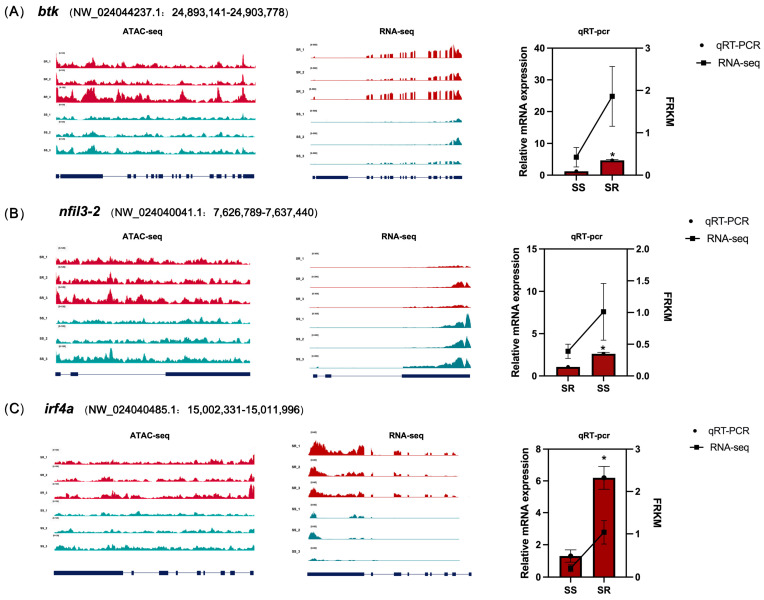
Validation results for (**A**–**C**) *btk*, *nfil3-2*, and *irf4a*, respectively. Left panels: Genome browser views showing ATAC-seq chromatin accessibility and RNA-seq transcript levels. Blue bars at the bottom indicate gene structures, highlighting differences in chromatin accessibility between the two groups. The vertical axis height represents the enrichment of sequencing reads, directly corresponding to the degree of accessibility. Right panels: Relative mRNA expression levels measured by qRT-PCR. Left Y-axis: relative expression (qRT-PCR); right Y-axis: Fragments Per Kilobase of transcript per Million mapped reads (FPKM) (RNA-Seq). Note that the two Y-axes have different scales. Data are shown as mean ± SEM; Note: * indicates significant difference between the SS group and the SR group (*p* < 0.05).

To verify the accuracy of the RNA-seq results, four differentially expressed transcription factors (CTCF, ETV4, ATF3, and ELK4) were randomly selected for qRT-PCR validation (Figure 7). The results showed that, compared with the SR group, the expression level of ETV4 was significantly decreased in the SS group (*p* < 0.05), while the expression levels of ATF3 and CTCF were significantly increased (*p* < 0.05). No significant difference was observed for ELK4. In the RNA-seq analysis, the expression levels of ETV4 and ATF3 in the SS group showed significant differences compared with the SR group, with log_2_FC values of −1.41 and 2.20, respectively (*p* < 0.05). The expression level of CTCF was higher in the SS group (log_2_FC = 0.45), while no significant difference was detected for ELK4 (log_2_FC = −0.04). The expression trends observed by qRT-PCR were consistent with those from RNA-seq, confirming the reliability of the transcriptome data.

The expression levels of the candidate genes *irf4a*, *btk*, and *nfil3-2*, predicted to be regulated by ETS and FOX family transcription factors, were further investigated by qRT-PCR. The results showed that the expression levels of *irf4a* and *btk* in the resistant group were significantly higher than those in the SS group (*p* < 0.05), being 4.77-fold and 3.79-fold higher, respectively, indicating that *irf4a* and *btk* may play key roles in enhancing disease resistance in tilapia. In contrast, the expression level of *nfil3-2* in the resistant group was significantly lower than that in the SS group (*p* < 0.05), showing a 2.45-fold difference, suggesting that *nfil3-2* may play an inhibitory role in the immune response. In the resistant group, the chromatin accessibility of the immune activation-related genes (*irf4a*, *btk*) was higher, consistent with their elevated expression levels. In contrast, although the chromatin accessibility of the immune-suppressive gene *nfil3-2* was also higher, its expression level was significantly reduced. Detailed information on these genes from ATAC-seq, RNA-seq, and qRT-PCR analyses is presented in Figure 7.

## 3. Discussion

In the host–virus battle, the innate immune response serves as the first line of defense against infection, with precise transcriptional regulation of immune-related genes at its core. During iridovirus infection, significant changes in open chromatin reflect the host cell’s ability to regulate gene expression at the chromatin level [32]. In this study, analysis of differentially accessible chromatin regions (DARs) between the SR and SS groups using ATAC-seq revealed that DAR-associated genes are widely involved in immune-related signaling pathways, including autophagy, apoptosis, immune cell differentiation, and the C-type lectin receptor, Toll-like receptor, and NOD-like receptor pathways. Similarly, in Nile tilapia infected with Streptococcus agalactiae [38], DAR-associated genes were also mainly enriched in immune-related pathways, including the Toll-like receptor pathway, lysosome, cell cycle, and endocytosis. Our study further found that the transcriptional activation of these immune-related genes is highly dependent on the chromatin state of their core promoter regions. The majority of promoter DARs in the SR group exhibited increased accessibility, suggesting that chromatin remodeling in these regions may be a key mechanism regulating the transcriptional activity of immune genes. As the critical region for RNA polymerase II transcription initiation complex assembly, the structure and function of the core promoter directly determine the efficiency, specificity, and dynamic responsiveness of gene expression. ATAC-seq analysis of the turbot head kidney infected with bacteria and viruses revealed that promoter-associated DARs were mostly immune-related [36]. Studies have also shown that peaks located in specific promoter regions play an important role in the expression of immune-responsive genes in Nile tilapia [38]. Collectively, this evidence points to the fact that promoter-associated DARs play a crucial role in immune regulation.

Transcription factors can precisely regulate the transcription of downstream immune effector genes by forming oligomers, thereby coordinating the host’s defense response against viral and bacterial pathogens [39,40]. This regulatory function relies on the structural diversity of transcription factor oligomers, with dimerization representing the most fundamental molecular form that generates such structural and functional specificity. In this study, we found that several of the key differentially expressed transcription factors exist as heterodimers, such as ERF::FOXI1, ETV2::FOXI1, and FOXJ2::ELF1. This indicates that the transcriptional regulation of antiviral immunity is not accomplished by individual transcription factors acting alone but rather through the cooperative interactions between members of different families. This cooperative regulatory model has important biological significance: at the dimer level, different subunit combinations can bind to specific DNA sequences in a highly specific manner, initiating distinct downstream signaling cascades and enabling the fine-tuning of immune responses [39,41]. Heterodimerization of transcription factors has been widely demonstrated to participate in various key physiological processes in fish. For example, in tongue sole (*Cynoglossus semilaevis*) [42], the hypoxia-inducible factor-1α (Hif-1α) of the basic helix-loop-helix (bHLH) family must form a heterodimer with the aryl hydrocarbon receptor nuclear translocator (Arnt) to effectively bind to the hypoxia response element (HRE) in the promoter regions of target genes, thereby activating downstream genes involved in energy metabolism reprogramming, angiogenesis, and erythropoiesis. In blunt snout bream (*Megalobrama amblycephala*) infected with *Aeromonas hydrophila* [43], the p50/p65 heterodimer of the nuclear factor-κB (NF-κB) family binds to κB sites in immune effector genes to initiate downstream immune responses. Carlos et al. [44] also found in zebrafish (*Danio rerio*) that two paralogs of Toll-like receptor 5 (TLR5), TLR5a and TLR5b, can form a heterodimer, recognize bacterial flagellin, activate the NF-κB signaling pathway, and induce the expression of pro-inflammatory genes. These findings collectively demonstrate that heterodimers play important roles in the growth, development, and reproduction of aquatic animals.

In this study, integrated analysis of ATAC-seq and RNA-seq data identified seven transcription factors (TFs) enriched in core promoter regions, six of which belong to the ETS or FOX families (Spi1, ETV2::FOXI1, FOXJ2::ELF1, FOXO1::ELK1, SPIC, and FOXO1::ELF1). ETS family TFs are primarily responsible for lymphocyte development and differentiation and are characterized by a highly conserved ETS DNA-binding domain [45]. Spi1 is a well-recognized immune-related transcription factor within the ETS family [46], often referred to as the “master regulator” of hematopoiesis, directing the differentiation of hematopoietic stem cells toward the myeloid and lymphoid lineages. High expression of Spi1 promotes macrophage differentiation, moderate expression drives B cell differentiation, and low expression leads to T cell differentiation [47]. Within the immune system, Spi1 activates B cell- and macrophage-specific genes, making it a key regulator of both adaptive and innate immunity [48]. SPIC belongs to the same subfamily as Spi1 and primarily functions in specific subsets of B cells and macrophages. It is involved in the generation and maintenance of marginal-zone B cells and regulates the expression of genes related to iron metabolism, contributing to the fine-tuning of immune tolerance [49]. ETV2 has been identified as a master regulator of cardiovascular system development. In the immune system, ETV2 may indirectly influence immune cell recruitment and migration by regulating vascular endothelial function [50]. ELF1 is involved in lymphocyte development and functional regulation. It plays a role in T cell activation and differentiation, regulating the expression of various immune effector genes [51]. ELK1, another ETS family member, primarily participates in downstream transcriptional regulation of the MAPK signaling pathway, coupling extracellular signals to changes in gene expression and playing roles in cell proliferation, differentiation, and stress responses [52]. FOX family TFs are mainly involved in cell cycle and metabolic regulation. In the fine flounder (*Paralichthys adspersus*), both FOXO1 and FOXO3 have been shown to mediate starvation-induced muscle atrophy [53]. These FOXO family members drive muscle wasting by upregulating *atrogin-1* and *MuRF1*—key components of the ubiquitin-proteasome system—thereby promoting protein degradation. Under stressful conditions or fasting in teleosts, inhibition of the IGF-1/Akt signaling pathway leads to enhanced FOXO activation, which then translocates to the nucleus and triggers the transcription of catabolic genes [54]. Thus, ETS family TFs are primarily responsible for immune cell differentiation and functional activation, while FOX family TFs maintain cellular homeostasis. Their cooperation may ensure an effective immune response that clears pathogens while avoiding excessive inflammatory damage.

Based on the ETS and FOX family transcription factors identified above, the expression trends of three key immune-related target genes (*irf4a*, *btk*, and *nfil3-2*) were further confirmed via qRT-PCR, which yielded results in agreement with the RNA-seq data. The expression levels of *irf4a* and *btk* were significantly increased in the SR group, while *nfil3-2* showed the opposite trend, indicating that changes in chromatin accessibility do not always correspond with gene expression levels. This phenomenon has been observed in previous studies. For example, in turbot experimentally infected with viruses and bacteria [36], some up-regulated DEGs (e.g., the transcription factor genes *egr1*, *meis1*, *mitf*) did not show significant changes in chromatin accessibility in their promoter regions. This suggests that these genes may be in a “poised” state, where their chromatin is already pre-opened at the basal level, allowing for rapid transcriptional responses without the need for further remodeling. Similarly, Jiao et al. [38] found in Nile tilapia infected with *Streptococcus agalactiae* that increased chromatin accessibility in the promoter region of the *birc2* gene did not lead to its up-regulation and even showed an opposite trend. These findings all indicate that the relationship between chromatin accessibility changes and gene expression is not simply linear. *Nfil3-2* is a teleost-specific paralog of NFIL3, and its regulatory function exhibits species-specific and environment-dependent characteristics. In grass carp (*Ctenopharyngodon idella*), *nfil3* can activate pro-inflammatory pathways following bacterial infection [55]. In mammals, NFIL3 typically acts as a transcriptional repressor, participating in the regulation of immunity, metabolism, and circadian rhythms [56,57,58]. In the present study, the expression of *nfil3-2* was significantly down-regulated in the SR group. Given that excessive inflammatory responses are often accompanied by tissue damage and immunopathology, the down-regulation of nfil3-2 in the SR group may help suppress excessive inflammatory responses, thereby playing a protective role in antiviral immunity. *Irf4a* is an important member of the IRF family. In blunt snout bream (*Megalobrama amblycephala*), *irf4a* is constitutively expressed in all tissues and is significantly up-regulated following Aeromonas hydrophila infection, suggesting its role in antibacterial immunity [59]. In common carp (*Cyprinus carpio*), IRF4 has been identified as a negative regulator of type I interferon (IFN) and NF-κB signaling pathways, fine-tuning immune responses by inhibiting these two key antiviral pathways to prevent excessive inflammatory damage [60]. Similarly, in yellow catfish (*Pelteobagrus fulvidraco*), three IRF4 paralogs have been confirmed to negatively regulate type I IFN responses [61]. *BTK* is a core kinase in the B cell receptor (BCR) signaling pathway. Studies have shown that in Asian seabass (*Lates calcarifer*) infected with red-spotted grouper nervous necrosis virus (RGNNV) [62], *BTK* is involved in BCR signaling and is crucial for B cell activation and function [63,64]. These results further demonstrate that heterodimers of ETS and FOX family transcription factors mediate a crucial role in the host immune reaction of largemouth bass upon LMBV challenge.

## 4. Materials and Methods

### 4.1. LMBV Virus Strain

Samples were collected from naturally infected largemouth bass at the Freshwater Fisheries Research Center, Chinese Academy of Fishery Sciences (FFRC, Wuxi, China). Individuals with typical clinical signs, including extensive body surface ulceration, redness and swelling at the fin bases, as well as pale liver, blackened spleen, and enlarged kidney upon dissection, were selected. Liver, spleen, and kidney tissues were aseptically collected and homogenized for virus isolation, purification, and subsequent culture. Additionally, an iridovirus nucleic acid detection kit (Guangzhou Double Helix Gene Technology Co., Ltd., Guangzhou, China) was used to confirm infection status.

The prepared tissue homogenate was subjected to three cycles of freezing at −80 °C and thawing at room temperature to fully release virus particles. After centrifugation at 4000 r/min for 30 min at 4 °C, the supernatant was collected and filtered through a 0.22 μm filter membrane. The resulting filtrate was aliquoted and stored at −80 °C as the virus stock solution. Virus amplification was performed using the Epithelioma papulosum cyprini (EPC) cell line as host cells [65]. A mixture of 200 μL of the virus stock solution and 800 μL of M199 medium containing 2% fetal bovine serum (FBS) was inoculated onto a healthy EPC cell monolayer. After adsorption at 25 °C for 1 h, 4 mL of M199 medium containing 2% FBS was added. The cell culture flask was then transferred to a 25 °C incubator with 5% CO_2_, and the development of cytopathic effects (CPEs) was observed daily. When approximately 80% of the cells exhibited typical CPE [66], the cell culture was collected and subjected to three additional freeze–thaw cycles. After centrifugation, the supernatant containing virus particles was collected. The virus titer (TCID_50_) was calculated using the Reed–Muench method [67], and the TCID_50_ of the LMBV virus stock obtained in this study was determined to be 4 × 10^5^/mL. The purified virus was stored at −80 °C for subsequent use.

### 4.2. Challenge Experiment and Tissue Collection

The experimental fish used in this study were derived from an F_3_ generation population of largemouth bass (mean body weight 19.5 ± 0.6 g, mean body length 12.1 ± 0.7 cm) selectively bred at the Freshwater Fisheries Research Center, Chinese Academy of Fishery Sciences. Preliminary experiments conducted by our research group indicated that immersion challenge with LMBV at a concentration of 40 TCID_50_/mL resulted in a cumulative infection rate of 50–60% in largemouth bass over a 28-day observation period [68]. Accordingly, this concentration was adopted as the formal challenge dose in the present experiment. Prior to the experiment, 30 individuals were randomly sampled and tested for LMBV to confirm negative status before commencement of the trial. All experimental fish were acclimated for two weeks in a holding tank (4.5 m × 8.8 m × 1.0 m) to allow adaptation to the experimental conditions. During the acclimation period, water temperature was maintained at 26 ± 0.5 °C, dissolved oxygen concentration remained above 6 mg/L, and water pH ranged from 7.2 to 7.6. Following acclimation, a total of 400 healthy individuals with normal feeding behavior and uniform size (mean body weight 21.0 ± 0.5 g, mean body length 12.3 ± 0.8 cm) were selected and transferred to an experimental tank (2.0 m × 4.0 m × 1.0 m). All experimental water was subjected to filtration, disinfection, and sufficient aeration prior to use, with water temperature maintained consistently at 26 ± 0.5 °C.

The iridovirus suspension was evenly sprayed onto the water surface of the experimental tank (2.0 m × 4.0 m × 1.0 m), with 800 mL of viral inoculum added per tank to achieve and maintain a final waterborne concentration of 40.0 TCID_50_/mL. During the experimental period, two-thirds of the culture water was renewed weekly, after which the viral suspension was reapplied, supplemented with approximately 267 mL of viral inoculum to restore the target concentration. The viral titer in the water column was monitored periodically to ensure its stability throughout the trial. Environmental parameters were rigorously controlled as follows: water temperature was maintained at 26 ± 0.5 °C, dissolved oxygen concentration was kept at ≥7.5 mg/L, and total ammonia nitrogen was maintained at ≤1 mg/L. Fish were fed a commercial diet twice daily at scheduled intervals (08:00 and 16:30), with the daily feeding rate adjusted to 3–5% of total body weight. Clinical signs were observed and recorded daily throughout the experimental period. At the termination of the experiment, cumulative mortality was calculated, and the survival rate and the number of infected individuals among the apparently healthy largemouth bass were confirmed using an iridovirus-specific nucleic acid detection kit.

A total of 200 experimental fish were randomly selected for the viral challenge trial. Mortality was initially observed between 12 and 24 h post-infection (hpi), and the survival time of each individual was meticulously recorded. To minimize potential bias associated with injection procedures, no sampling was conducted on individuals that succumbed within the first 48 hpi. Mortality was defined as the absence of spontaneous movement, cessation of opercular activity, and lack of response to external tactile stimuli [69]. During the peak mortality window occurring between 48 and 72 hpi, splenic tissues were aseptically collected from moribund fish to constitute the susceptible group (SS). Conversely, splenic tissues obtained from individuals that survived beyond five weeks post-challenge and exhibited no overt clinical signs of disease were assigned to the resistant group (SR). Specifically, on day 3 and day 35 post-challenge, six splenic tissue samples were aseptically harvested from each of the SS and SR groups, respectively. These six samples were subsequently pooled pairwise to generate three composite biological replicates per group, which were designated for downstream ATAC-seq and RNA-seq analyses. Additionally, three separate splenic tissue samples were collected from each group and preserved for histopathological examination.

### 4.3. Histological Analysis

Splenic tissue samples collected for histopathological examination were immersed in Bouin’s fixative and fixed for 24 h at 4 °C. Following fixation, tissues were repeatedly rinsed with 70% ethanol to thoroughly remove residual fixative and subsequently stored in 70% ethanol until further processing. The specimens were dehydrated through a graded ascending ethanol series to progressively displace tissue water, followed by clearing in xylene. The cleared tissues were infiltrated and embedded in paraffin wax using an automated embedding station. Once the paraffin blocks had fully solidified, serial sections were cut at a thickness of 7 μm using a rotary microtome. Tissue sections mounted on glass slides were deparaffinized in xylene and rehydrated through a graded descending ethanol series to an aqueous phase. Rehydrated sections were stained with hematoxylin solution for 3–5 min to visualize nuclear components, followed by counterstaining with eosin solution for 5 min to delineate cytoplasmic structures and extracellular matrix. Following the staining procedure, sections were dehydrated through an ascending ethanol series, cleared in xylene, and mounted with neutral balsam mounting medium. The prepared histological slides were examined under a Nikon Eclipse Ci-L light microscope (Nikon Corporation, Tokyo, Japan) equipped with a digital imaging system. Micrographs were captured, and splenic cytoarchitectural alterations and pathological characteristics were systematically documented and analyzed.

### 4.4. ATAC-Seq Data Analysis

Raw sequencing data quality was initially assessed using FastQC (version 0.11.9), and low-quality reads were filtered using fastp (version 0.19.5; https://github.com/OpenGene/fastp (accessed on 27 April 2026)) with default parameters to remove adapter contamination and trim bases with Phred quality scores below 20. The resulting clean reads were aligned to the largemouth bass reference genome (assembly accession: GCF_014851395.1) using Bowtie2 (version 2.2.8) with the parameter-X 2000 to specify the maximum fragment length for valid paired-end alignments. Reads mapping to the mitochondrial genome were discarded, and only uniquely mapped reads with high alignment confidence were retained for subsequent downstream analyses. To assess the global distribution of chromatin accessibility surrounding transcriptional regulatory regions, the deepTools suite (version 3.2.0) was employed to compute the average read coverage depth across all annotated transcription start sites (TSSs) within a ±2 kb flanking window. Read counts were aggregated in non-overlapping 50 bp bins and normalized to generate coverage profiles. Peak calling to identify regions of significantly enriched open chromatin was performed using MACS2 (version 2.1.2) with ATAC-seq-specific parameters: --nomodel --shift -100 --extsize 200 -*p* 0.01. Peaks meeting the significance threshold were retained for further analysis. Genomic annotation of identified peaks was conducted using the R/Bioconductor package ChIPseeker (version 1.16.1), wherein peaks were assigned to the nearest gene feature. During whole-genome peak annotation, functional genomic regions were systematically classified into discrete categories, including promoter regions, downstream regions, exons, introns, and distal intergenic regions. Furthermore, peak enrichment profiles across TSS regions were visualized to validate assay specificity. Differential chromatin accessibility analysis between the resistant (SR) group and the susceptible (SS) group was performed using the R/Bioconductor package DiffBind (version 2.8.0). Statistically significant differentially accessible peaks were identified based on stringent criteria of |log_2_ fold change| ≥ 1 and a false discovery rate (FDR) adjusted *p*-value ≤ 0.05. Genes associated with these differentially accessible regions (DARs) were subsequently annotated. Finally, transcription factor binding motif discovery was conducted on the differentially accessible peak sets using the Multiple Expectation maximizations for Motif Elicitation suite (MEME Suite) to predict conserved motifs and potential regulatory elements enriched within these open chromatin intervals. To further explore the potential regulatory targets of these transcription factors, the Find Individual Motif Occurrences (FIMO) tool was used to scan for TF binding positions within the core promoter region (from 50 bp upstream to 50 bp downstream of the transcription start site, TSS). Genes containing these binding sites were annotated as downstream target genes.

### 4.5. RNA-Seq Data Analysis

Quality control and adapter trimming of raw Illumina sequencing reads were performed using fastp (version 0.18.0) [49]. The resulting high-quality clean reads were subsequently aligned to the largemouth bass reference genome (GCF_014851395.1) using the splice-aware aligner HISAT2 (version 2.1.0). Based on the alignment outputs, transcript assembly and abundance estimation were performed using StringTie10), and gene-level expression counts were quantified for each sample using RSEM (RNA-Seq by Expectation-Maximization; version 1.2.19). The resulting count matrix was imported into the R statistical environment, and differential expression analysis between the resistant (SR) and susceptible (SS) groups was conducted using the DESeq2 package (version 1.6.1) within the Bioconductor framework. Genes exhibiting an absolute log_2_ fold change (|log_2_FC|) ≥ 1 and a Benjamini–Hochberg adjusted false discovery rate (FDR) < 0.05 were considered significantly differentially expressed (DEGs).

### 4.6. Functional Enrichment Analysis

To characterize the functional implications of genes associated with DARs and DEGs, functional enrichment analyses were performed separately for each gene set. Gene Ontology (GO) enrichment analysis was conducted using the GOseq R package (version 1.46.0), which accounts for gene length bias inherent in RNA-seq data. Concurrently, KOBAS 2.0 software was employed for pathway enrichment analysis by mapping genes to the Kyoto Encyclopedia of Genes and Genomes (KEGG) database, thereby enabling the identification of significantly altered biological functions and key signaling cascades. Furthermore, to identify coordinated transcriptional programs and pathway-level alterations that may not be captured solely by individual DEG thresholds, Gene Set Enrichment Analysis (GSEA) was applied to the RNA-seq dataset using GSEA software (version 2.2.4). All detected genes were preranked based on the Signal2Noise metric to evaluate the distribution trend of predefined gene sets within the ranked list. An Enrichment Score (ES) was calculated to reflect the degree of overrepresentation of a gene set at the extremes of the ranked phenotype. To account for variations in gene set size and to assess statistical significance, the ES was normalized to a Normalized Enrichment Score (NES) via permutation testing. Gene sets exhibiting an NES absolute value ≥ 1.5 and a false discovery rate (FDR) ≤ 0.25 were considered significantly enriched.

### 4.7. Quantitative Real-Time PCR Validation

Quantitative real-time PCR (qRT-PCR) was employed to assess the mRNA expression profiles of immune-related genes in the spleen of largemouth bass following LMBV infection. Total RNA was extracted from splenic tissue samples using the VAMNE Magnetic Pathogen RNA Kit (Vazyme, Nanjing, China) in conjunction with the VNP-96P Automated Nucleic Acid Extraction System. RNA integrity and purity were examined by electrophoresis on a 1% agarose gel and spectrophotometric analysis using a NanoDrop spectrophotometer (Thermo Fisher Scientific, Waltham, MA, USA). To eliminate potential genomic DNA contamination, RNA samples were treated with RNase-free DNase. First-strand complementary DNA (cDNA) was subsequently synthesized from the purified RNA using the miRNA 1st Strand cDNA Synthesis Kit (Vazyme, Nanjing, China) according to the manufacturer’s instructions. Quantitative real-time PCR amplification was performed on an ABI QuantStudio 5 Real-Time PCR System (Applied Biosystems, Foster City, CA, USA) using SYBR^®^ Premix Ex Taq™ Kit (Takara Bio Inc., Dalian, China). Each 20 μL reaction mixture consisted of 10 μL of 2 × SYBR Premix Ex Taq™, 1 μL of each forward and reverse primer, 2 μL of cDNA template, and RNase-free dH_2_O to volume. The thermal cycling protocol was configured as follows: initial denaturation at 95 °C for 5 min, followed by 40 cycles of denaturation at 95 °C for 5 s, annealing at 60 °C for 10 s, and extension at 72 °C for 15 s. All qRT-PCR assays were conducted with three independent biological replicates and three technical replicates for each sample. The 2^−ΔΔCt^ method [70] served to compute relative expression levels of genes and normalized to the expression of the reference gene *β-actin*. The sequences of all primers utilized in this study are listed in Table 1.

### 4.8. Data Integration Analysis

SPSS (version 27.0; IBM Corp., Armonk, NY, USA) was employed for all statistical analyses in the present study. Comparisons between two independent groups were performed using the independent-samples Student’s *t*-test, with statistical significance set at *p* < 0.05. All experimental data are presented as the mean ± standard deviation (Mean ± SD) of at least three independent biological replicates, unless otherwise specified. Graphical representation of data and generation of figures were carried out using GraphPad Prism (version 9.5.0; GraphPad Software, San Diego, CA, USA) and OriginPro 2024 (OriginLab Corporation, Northampton, MA, USA). Genomic signal tracks and chromatin accessibility peaks were visualized using the Integrative Genomics Viewer (IGV; Broad Institute, Cambridge, MA, USA).

## 5. Conclusions

In the present study, integrated analysis of ATAC-seq and RNA-seq was employed to delineate, for the first time, the dynamic chromatin accessibility landscape associated with resistance to LMBV infection in largemouth bass. Key transcription factors and their corresponding target genes intimately involved in the regulation of the antiviral immune response were successfully identified. Further mechanistic investigation revealed that chromatin remodeling and enhanced accessibility within core promoter regions potentiated the synergistic regulatory capacity of ETS/FOX family transcription factor heterodimers on immune-related genes. This coordinated transcriptional activation ultimately culminates in the establishment of an effective antiviral immune response mediated by downstream effector target genes, including *irf4a*, *btk*, and *nfil3-2*.

## Figures and Tables

**Table 1:** Primer sequence for qRT-PCR.

Gene Full Name	Primer Names	Sequence (5′-3′)
CCCTC-binding factor	CTCF-F′	CTCTGCTTTCGGCCGAGTTA
	CTCF-R′	TGGGTAGGAACTCTTTGTGGTG
ETS variant transcription factor 4	ETV4-F′	CAGCAGCATTACTCCCCGAA
	ETV4-R′	TGGCAGCAGGGTTCATGTAG
activating transcription factor 3	ATF3-F′	CGGGCTCAGTGCTGACATAA
	ATF3-R′	CTCCTGATCCACAGGCAAGG
ETS transcription factor ELK4	ELK4-F′	GCCATGGAGCCAACCCTAAT
	ELK4-R′	CCGATGGGCGGATATGTTCA
*interferon regulatory factor 4a*	*irf4a*-F′	GAAGGAGCAGCCATGCAAAC
	*irf4a*-R′	GTCTCGGTAAATGTCGGCCA
*Bruton agammaglobulinemia tyrosine kinase*	*btk*-F′	CGAGCAACTGAGGCAAAGGT
	*btk*-R′	TGCAGATGCTGAGGCTTGAA
*nuclear factor, interleukin 3 regulated, member 2*	*nfil3-2*-F′	CACTGGAGACAAGCGACACT
	*nfil3-2*-R′	GTCCAAGAATCCGCTTTGCG
*β-ACTIN*	*β-ACTIN*-F′	CCACCACAGCCGAGAGGGAA
	*β-ACTIN*-R′	TCATGGTGGATGGGGCCAGG

## Data Availability

All datasets produced or examined in the present work can be found within the main article and the accompanying Supplementary Materials. The RNA-seq and ATAC-seq data generated in this study have been submitted to the NCBI Gene Expression Omnibus (GEO). The RNA-seq data can be found under BioProject accession PRJNA1457692, and the ATAC-seq data under BioProject accession PRJNA1457840.

## Supplementary Materials

The following supporting information can be downloaded at: https://www.mdpi.com/article/10.3390/ijms27094124/s1.

## References

[1] Hussein G.H.G., Chen M., Qi P.P., Cui Q.K., Yu Y., Hu W.H., Tian Y., Fan Q.X., Gao Z.X., Feng M.W. (2020). Aquaculture industry development, annual price analysis and out-of-season spawning in largemouth bass *Micropterus salmoides*. Aquaculture.

[2] Bai J.J., Lutz-Carrillo D.J., Quan Y.C., Liang S.X. (2008). Taxonomic status and genetic diversity of cultured largemouth bass *Micropterus salmoides* in China. Aquaculture.

[3] Fisheries Administration Bureau of the Ministry of Agriculture and Rural Affairs, National Station for Aquatic Technology Extension, Chinese Society of Fisheries (2024). 2024 China Fisheries Statistical Yearbook.

[4] Deng G.C., Li S.J., Xie J., Bai J.J., Chen K.C., Ma D.M., Jiang X.Y., Lao H.H., Yu L.Y. (2011). Characterization of a ranavirus isolated from cultured largemouth bass (*Micropterus salmoides*) in China. Aquaculture.

[5] Fogelson S.B., Petty B.D., Reichley S.R., Ware C., Bowser P.R., Crim M.J., Getchell R.G., Sams K.L., Marquis H., Griffin M.J. (2016). Histologic and molecular characterization of Edwardsiella piscicida infection in largemouth bass (*Micropterus salmoides*). J. Vet. Diagn. Investig..

[6] Jiang B., Lu G.L., Du J.J., Wang J., Hu Y.Z., Su Y.L., Li A.X. (2019). First report of trypanosomiasis in farmed largemouth bass (*Micropterus salmoides*) from China: Pathological evaluation and taxonomic status. Parasitol. Res..

[7] Zhang M.J., Chen X.Y., Xue M.Y., Jiang N., Li Y.Q., Fan Y.D., Zhang P., Liu N.C., Xiao Z.D., Zhang Q.H. (2023). Oral vaccination of largemouth bass (*Micropterus salmoides*) against largemouth bass ranavirus (LMBV) using yeast surface display technology. Animals.

[8] Zhu C.K., Liu D., Wang W.J., Li Y., Li Z.X., He H., He B.W., Zhu L., Chu P.F. (2024). Pathogenicity characterization, immune response mechanisms, and antiviral strategies analysis underlying a LMBV strain in largemouth bass (*Micropterus salmoides*). Aquac. Rep..

[9] Wang R.X., Luo X., Li N.Q., Lin Q., Niu Y.J., Liang H.R., Fu X.Z., Lv A.J. (2024). Preparation and application of polyclonal antibody against MCP protein of largemouth bass ranavirus. J. Northwest A F Univ. (Nat. Sci. Ed.).

[10] Liu X.D., Zhang L.W., Tan X., Guo M.Y., Kong W.G., An Z.H. (2024). MicroRNA profiling yields immune response and metabolic changes in juvenile largemouth bass (*Micropterus salmoides*) infected with LMBV. Water Biol. Secur..

[11] Mao J., Wang J., Chinchar G.D., Chinchar V.G. (1999). Molecular characterization of a ranavirus isolated from largemouth bass *Micropterus salmoides*. Dis. Aquat. Org..

[12] Zhao R.X., Geng Y., Qin Z.Y., Wang K.Y., Ouyang P., Chen D.F., Huang X.L., Zuo Z.C., He C.L., Guo H.R. (2020). A new ranavirus of the Santee-Cooper group invades largemouth bass (*Micropterus salmoides*) culture in southwest China. Aquaculture.

[13] Mondal H., Thomas J. (2022). A review on the recent advances and application of vaccines against fish pathogens in aquaculture. Aquac. Int..

[14] Xu C., Li E.C., Suo Y.T., Su Y.J., Lu M.H., Zhao Q., Qin J.G., Chen L.Q. (2018). Histological and transcriptomic responses of two immune organs, the spleen and head kidney, in Nile tilapia (*Oreochromis niloticus*) to long-term hypersaline stress. Fish Shellfish Immunol..

[15] Ellis E.F., Smith R.T. (1966). The role of the spleen in immunity: With special reference to the post-splenectomy problem in infants. Pediatrics.

[16] Xia Y.C., Cao Z., Lin L.Y., Pan X.Y., Yao J.Y., Liu Y.H., Yin W.L., Shen J.Y. (2018). Research progress on main diseases of largemouth bass (*Micropterus salmoides*). China Anim. Health Insp..

[17] Vendramin N., Alencar A.L.F., Iburg T.M., Dahle M.K., Wessel Ø., Olsen A.B., Rimstad E., Olesen N.J. (2018). Piscine orthoreovirus infection in Atlantic salmon (*Salmo salar*) protects against subsequent challenge with infectious hematopoietic necrosis virus (IHNV). Vet. Res..

[18] Wang Q., Wang S.W., Zhang Y., Yu Y.P., Zhao H.H., Yang H.R., Zheng L.Y., Yang M., Qin Q.W. (2019). The CXC chemokines and CXC chemokine receptors in orange-spotted grouper (*Epinephelus coioides*) and their expression after Singapore grouper iridovirus infection. Dev. Comp. Immunol..

[19] Chavanne H., Janssen K., Hofherr J., Contini F., Haffray P., Komen H., Nielsen E.E., Bargelloni L. (2016). A comprehensive survey on selective breeding programs and seed market in the European aquaculture fish industry. Aquac. Int..

[20] Li P.H., Luo X., Zuo S.Z., Fu X.Z., Lin Q., Niu Y.J., Liang H.R., Ma B.F., Li N.Q. (2024). Genome-wide association study of resistance to largemouth bass ranavirus (LMBV) in *Micropterus salmoides*. Int. J. Mol. Sci..

[21] Sahoo P.K., Meher P.K., Mahapatra K.D., Saha J.N., Jana R.K., Reddy P.V.G.K. (2004). Immune responses in different fullsib families of Indian major carp, *Labeo rohita*, exhibiting differential resistance to *Aeromonas hydrophila* infection. Aquaculture.

[22] Camp K.L., Wolters W.R., Rice C.D. (2000). Survivability and immune responses after challenge with *Edwardsiella ictaluri* in susceptible and resistant families of channel catfish, *Ictalurus punctatus*. Fish Shellfish Immunol..

[23] Xiong X.M., Chen Y.L., Liu L.F., Wang W.M., Robinson N.A., Gao Z.X. (2017). Estimation of genetic parameters for resistance to *Aeromonas hydrophila* in blunt snout bream (*Megalobrama amblycephala*). Aquaculture.

[24] Hirschhorn J.N., Daly M.J. (2005). Genome-wide association studies for common diseases and complex traits. Nat. Rev. Genet..

[25] Mendel J., Jánová K., Palíková M. (2018). Genetically influenced resistance to stress and disease in salmonids in relation to present-day breeding practice–a short review. Acta Vet. Brno.

[26] Xu W.H., Zhang Z.M., Lai F.X., Yang J.H., Qin Q.W., Huang Y.H., Huang X.H. (2023). Transcriptome analysis reveals the host immune response upon LMBV infection in largemouth bass (*Micropterus salmoides*). Fish Shellfish Immunol..

[27] Sun H., Hua J.X., Tao Y.F., He J., Wang Q.C., Dong Y.L., He J.X., Qiang J. (2025). Disease resistance and genetic characteristics of genetically selected largemouth bass (*Micropterus salmoides*) revealed by whole genome resequencing. Fish Shellfish Immunol..

[28] Xu Z.Q., Liao J.M., Zhang D.Z., Liu S.L., Zhang L.H., Kang S.Z., Xu L.T., Chen H., Peng W.Q., Zhou S. (2023). Isolation, characterization, and transcriptome analysis of an ISKNV-like virus from largemouth bass. Viruses.

[29] Wu Y.L., Lin Z.J., Li C.C., Lin X., Shan S.K., Guo B., Zheng M.H., Li F.X., Yuan L.Q., Li Z.H. (2023). Epigenetic regulation in metabolic diseases: Mechanisms and advances in clinical study. Signal Transduct. Target. Ther..

[30] Luo L.H., Gribskov M., Wang S.F. (2022). Bibliometric review of ATAC-Seq and its application in gene expression. Brief. Bioinform..

[31] Thurman R.E., Rynes E., Humbert R., Vierstra J., Maurano M.T., Haugen E., Sheffield N.C., Stergachis A.B., Wang H., Vernot B. (2012). The accessible chromatin landscape of the human genome. Nature.

[32] Klemm S.L., Shipony Z., Greenleaf W.J. (2019). Chromatin accessibility and the regulatory epigenome. Nat. Rev. Genet..

[33] Buenrostro J.D., Giresi P.G., Zaba L.C., Chang H.Y., Greenleaf W.J. (2013). Transposition of native chromatin for fast and sensitive epigenomic profiling of open chromatin, DNA-binding proteins and nucleosome position. Nat. Methods.

[34] Wapinski O.L., Lee Q.Y., Chen A.C., Li R., Corces M.R., Ang C.E., Treutlein B., Xiang C.M., Baubet V., Suchy F.P. (2017). Rapid chromatin switch in the direct reprogramming of fibroblasts to neurons. Cell Rep..

[35] Kong X.S., Wei G.S., Chen N., Zhao S.D., Shen Y.W., Zhang J.J., Li Y., Zeng X.Q., Wu X.F. (2020). Dynamic chromatin accessibility profiling reveals changes in host genome organization in response to baculovirus infection. PLoS Pathog..

[36] Aramburu O., Pardo B.G., Villamayor P.R., Hortas A.B., Lamas J., Dewari P., Morata D.P., Boudinot P., Macqueen D.J., Bouza C. (2025). Multiomics uncovers the epigenomic and transcriptomic response to viral and bacterial stimulation in turbot. GigaScience.

[37] Song C.W., Huang Y., Han F., Wang Z.Y. (2025). Chromatin accessibility and differentially expressed genes profiling in large yellow croaker (*Larimichthys crocea*) head kidney cells following iridovirus infection. Front. Immunol..

[38] Jiao J.P., Qiao T.F., Huang D.D., Zhu Z.X., Liu T.D., Xia J.H. (2025). Genome-wide chromatin accessibility profiles in spleen of GIFT strain of Nile tilapia (*Oreochromis niloticus*) in response to *Streptococcus agalactiae* infection as revealed by ATAC-seq and RNA-seq. Aquaculture.

[39] Ptashne M. (1986). A Genetic Switch: Gene Control and Phage λ.

[40] Potoyan D.A., Bueno C., Zheng W.H., Komives E.A., Wolynes P.G. (2017). Resolving the NFκB hetero-dimer binding paradox: Strain and frustration guide the binding of dimeric transcription factors. J. Am. Chem. Soc..

[41] Mazumdar P.M.H. (2003). The unfit: A history of a bad idea (review). Bull. Hist. Med..

[42] Qi Z.T., Wang Z.S., Zhang Q.H., Zhao W.H., Qiu M., Peng J.Q. (2013). Molecular cloning and expression analysis of hypoxia inducible factor 1α in tongue sole, *Cynoglossus semilaevis* (*Actinopterygii*: *Pleuronectiformes*: *Cynoglossidae*), subjected to acute hypoxia. Acta Ichthyol. Piscat..

[43] Wang S.J., Tai Z.P., Sun Q.H., Wang J.X., Wang H.L., Gao Z.X., Liu H. (2023). Akirin2 plays an important role in protecting *Megalobrama amblycephala* from *Aeromonas hydrophila* infection. Aquaculture.

[44] Voogdt C.G.P., Wagenaar J.A., van Putten J.P.M. (2018). Duplicated TLR5 of zebrafish functions as a heterodimeric receptor. Proc. Natl. Acad. Sci. USA.

[45] Sizemore G.M., Pitarresi J.R., Balakrishnan S., Ostrowski M.C. (2017). The ETS family of oncogenic transcription factors in solid tumours. Nat. Rev. Cancer.

[46] Gallant S., Gilkeson G. (2006). ETS transcription factors and regulation of immunity. Arch. Immunol. Ther. Exp..

[47] DeKoter R.P., Singh H. (2000). Regulation of B lymphocyte and macrophage development by graded expression of PU.1. Science.

[48] Zakrzewska A., Cui C., Stockhammer O.W., Benard E.L., Spaink H.P., Meijer A.H. (2010). Macrophage-specific gene functions in Spi1-directed innate immunity. Blood.

[49] Zhao C.L., Li Y.B., Tang J.L., Zhou Q.X., Lin X., Wen Z.L. (2023). Metaphocytes are IL-22BP-producing cells regulated by ETS transcription factor Spic and essential for zebrafish barrier immunity. Cell Rep..

[50] Sun N.N., Chu B., Choi D.H., Lim L., Song H. (2023). ETV2 enhances CXCL5 secretion from endothelial cells, leading to the promotion of vascular smooth muscle cell migration. Int. J. Mol. Sci..

[51] Tan Q.L., Wang J., Hao Y.M., Yang S.Z., Cao B., Pan W.J., Cao M.Y. (2025). Elf1 deficiency impairs macrophage development in zebrafish model organism. Int. J. Mol. Sci..

[52] Kalampounias G., Androutsopoulou T., Katsoris P. (2025). Mechanistic insights and clinical implications of ELK1 in solid tumors: A narrative review. Cells.

[53] Valenzuela C.A., Escobar D., Perez L., Zuloaga R., Estrada J.M., Mercado L., Valdés J.A., Molina A. (2015). Transcriptional dynamics of immune, growth and stress related genes in skeletal muscle of the fine flounder (*Paralichthys adpersus*) during different nutritional statuses. Dev. Comp. Immunol..

[54] Torres-Velarde J., Llera-Herrera R., García-Gasca T., García-Gasca A. (2018). Mechanisms of stress-related muscle atrophy in fish: An ex vivo approach. Mech. Dev..

[55] Yu H.Y., Shen Y.B., Sun J.L., Xu X.Y., Wang R.Q., Xuan Y.F., Lu L.Q., Li J.L. (2014). Molecular cloning and functional characterization of the NFIL3/E4BP4 transcription factor of grass carp, *Ctenopharyngodon idella*. Dev. Comp. Immunol..

[56] Qiang F.Y., Xuan D., Liu J.H., Sheng J. (2024). Knockdown of NFIL3 modulates the AMPK pathway to suppress excessive cell proliferation, inflammation, and migration in rheumatoid arthritis. Int. J. Rheum. Dis..

[57] Kashiwada M., Cassel S.L., Colgan J.D., Rothman P.B. (2011). NFIL3/E4BP4 controls type 2 T helper cell cytokine expression. EMBO J..

[58] Lin K.H., Kuo C.H., Kuo W.W., Ho T.J., Pai P., Chen W.K., Pan L.F., Wang C.C., Padma V.V., Huang C.Y. (2015). NFIL3 suppresses hypoxia-induced apoptotic cell death by targeting the insulin-like growth factor 2 receptor. J. Cell. Biochem..

[59] Zhan F.B., Jakovlić I., Wang W.M. (2019). Identification, characterization and expression in response to *Aeromonas hydrophila* challenge of five interferon regulatory factors in *Megalobrama amblycephala*. Fish Shellfish Immunol..

[60] Zhu Y.Y., Yang G.W. (2022). Molecular identification and functional characterization of IRF4 from common carp (*Cyprinus carpio* L.) in immune response: A negative regulator in the IFN and NF-κB signalling pathways. BMC Vet. Res..

[61] Tang Y.H., Lv X., Liu X.X., Song J.J., Wu Y.Q., Zhou Q., Zhu R. (2022). Three IRF4 paralogs act as negative regulators of type I IFN responses in yellow catfish (*Pelteobagrus fulvidraco*). Fish Shellfish Immunol..

[62] Angsujinda K., Plaimas K., Smith D.R., Kettratad J., Assavalapsakul W. (2020). Transcriptomic analysis of red-spotted grouper nervous necrosis virus infected Asian seabass *Lates calcarifer* (Bloch, 1790). Aquac. Rep..

[63] Mo Z.Q., Han Q., Zeng Y.L., Wang J.L., Li X.Z., Li Y.W., Sun H.Y., Li A.X., Luo X.C., Dan X.M. (2018). Molecular characterization and function analysis of grouper (*Epinephelus coioides*) Bruton’s tyrosine kinase BTK. Fish Shellfish Immunol..

[64] Nafez S., Oikawa K., Odero G.L., Sproule M., Ge N., Schapansky J., Abrenica B., Hatherell A., Cadonic C., Zhang S.Z. (2015). Early growth response 2 (Egr-2) expression is triggered by NF-κB activation. Mol. Cell. Neurosci..

[65] Fijan N., Sulimanović D., Bearzotti M., Muzinić D., Zwillenberg L.O., Chilmonczyk S., Vautherot J.F., de Kinkelin P. (1983). Some properties of the *Epithelioma papulosum cyprini* (EPC) cell line from carp *Cyprinus carpio*. Ann. Inst. Pasteur Virol..

[66] Wang T.E., Chao T.L., Tsai H.T., Lin P.H., Tsai Y.L., Chang S.Y. (2020). Differentiation of cytopathic effects (CPE) induced by influenza virus infection using deep convolutional neural networks (CNN). PLoS Comput. Biol..

[67] Reed L.J., Muench H. (1938). A simple method of estimating fifty per cent endpoints. Am. J. Hyg..

[68] Sun H., Hua J.X., Tao Y.F., Yang Z.Y., Zhu T.D., Lu S.Q., Wang W., Dong Y.L., Zhang L.B., He J.X. (2025). Development of a comprehensive lesion severity classification model for largemouth bass (*Micropterus salmoides*) ranavirus (LMBV) based on machine vision. Int. J. Mol. Sci..

[69] Galkanda-Arachchige H.S.C., Davis R.P., Nazeer S., Ibarra-Castro L., Davis D.A. (2021). Effect of salinity on growth, survival, and serum osmolality of red snapper, *Lutjanus campechanus*. Fish Physiol. Biochem..

[70] Zhang J.J., Li Y.Q., Zhou Y., Jiang N., Fan Y.D., Lin G., Zeng L.B. (2022). Characterization, expression pattern and antiviral activities of oligoadenylate synthetase in Chinese giant salamander, *Andrias davidianus*. Dev. Comp. Immunol..

