# Supplementary material for: An Integrative ATAC-Seq and RNA-Seq Analysis of Spleen Tissues from Largemouth Bass (Micropterus salmoides) Infected with Iridovirus (LMBV)
Source: Int J Mol Sci. 2026 May 5;27(9):4124. doi: 10.3390/ijms27094124 (PMC13164169; doi:10.3390/ijms27094124)
Supplement: Supplementary file 1 [file ijms-27-04124-s001.zip › supplement file/Supplemental table.docx]

Table S1. Summary of the ATAC-seq data in spleens of Largemouth Bass Infected with LMBV

| Sample | Clean Reads | Adapter(%) | N(%) | Mapped Reads | Duplicate Reads | Peaks Number | Consistent peaks Number |
| --- | --- | --- | --- | --- | --- | --- | --- |
| SR_1 | 129413782 | 7194(0.01%) | 166580(0.06%) | 0.9698 | 0.2589 | 33732 | 30027 |
| SR_2 | 118242420 | 7466(0.01%) | 117908(0.05%) | 0.9745 | 0.2544 | 38616 | 30027 |
| SR_3 | 127779564 | 7056(0.01%) | 124728(0.05%) | 0.9749 | 0.2875 | 43278 | 30027 |
| SS_1 | 102903182 | 6130(0.01%) | 181916(0.09%) | 0.9606 | 0.2155 | 12196 | 12077 |
| SS_2 | 100364204 | 6634(0.01%) | 202948(0.1%) | 0.9633 | 0.2473 | 17623 | 12077 |
| SS_3 | 168473054 | 10102(0.01%) | 193936(0.06%) | 0.9768 | 0.2636 | 25167 | 12077 |
| Total | 747176206 |  |  |  |  |  |  |

Table S2. All differential peaks between SS and SR groups

Due to the large size of the table, please refer to the accompanying Excel file (Supplementary Table S2).

Table S3. All significant differential peaks between SS and SR groups

Due to the large size of the table, please refer to the accompanying Excel file (Supplementary Table S3).

Table S4. The top 10 DARs located in core promoter regions

| peak_name | log2fc | p-value | FDR | anno_GeneId | Symbol |
| --- | --- | --- | --- | --- | --- |
| MergePeak_16021 | 2.17 | 2.23×10^-14^ | 5.88×10^-11^ | ncbi_119912453 | alpk1 |
| MergePeak_11085 | 2.00 | 9.15×10^-14^ | 1.31×10^-10^ | ncbi_119905425 | LOC119905425 |
| MergePeak_7800 | 1.94 | 8.97×10^-13^ | 6.91×10^-10^ | ncbi_119902946 | LOC119902946 |
| MergePeak_23130 | 2.16 | 1.13×10^-12^ | 8.08×10^-10^ | ncbi_119886081 | LOC119886081 |
| MergePeak_20203 | 2.26 | 2.05×10^-12^ | 1.21×10^-9^ | ncbi_119917015 | LOC119917015 |
| MergePeak_14159 | 1.99 | 2.26×10^-12^ | 1.30×10^-9^ | ncbi_119908934 | LOC119908934 |
| MergePeak_30344 | 2.19 | 9.40×10^-12^ | 3.44×10^-9^ | ncbi_119895771 | LOC119895771 |
| MergePeak_10951 | 2.51 | 9.46×10^-12^ | 3.44×10^-9^ | ncbi_119905349 | LOC119905349 |
| MergePeak_4115 | 1.99 | 1.81×10^-11^ | 5.34×10^-9^ | ncbi_119897533 | myh14 |
| MergePeak_18796 | 1.80 | 3.10×10^-11^ | 8.03×10^-9^ | ncbi_119915462 | LOC119915462 |
| MergePeak_14504 | 1.81 | 3.15×10^-11^ | 8.09×10^-9^ | ncbi_119910320 | LOC119910320 |
| MergePeak_7105 | 1.87 | 3.30×10^-11^ | 8.34×10^-9^ | ncbi_119900724 | calr |
| MergePeak_29474 | 1.83 | 3.38×10^-11^ | 8.41×10^-9^ | ncbi_119893129 | fam107b |
| MergePeak_27571 | 1.73 | 5.85×10^-11^ | 1.29×10^-8^ | ncbi_119891440 | scarf2 |
| MergePeak_25126 | 1.97 | 6.27×10^-11^ | 1.37×10^-8^ | ncbi_119888785 | egr1 |
| MergePeak_6712 | 1.89 | 6.64×10^-11^ | 1.43×10^-8^ | ncbi_119900436 | fcho1 |
| MergePeak_1188 | 2.18 | 6.77×10^-11^ | 1.45×10^-8^ | ncbi_119910509 | LOC119910509 |
| MergePeak_23858 | 1.62 | 8.28×10^-11^ | 1.69×10^-8^ | ncbi_119886232 | cdkn1a |
| MergePeak_9025 | 1.79 | 9.31×10^-11^ | 1.86×10^-8^ | ncbi_119902745 | LOC119902745 |
| MergePeak_4055 | 2.15 | 1.04×10^-10^ | 2.03×10^-8^ | ncbi_119897470 | zgc:92606 |

Table S5. Summary of the RNA-seq data in spleens of Largemouth Bass Infected with LMBV

| Sample | Clean Data(%) | Unique Mapped(%) | Adapter(%) | N(%) | Multiple Mapped(%) | Total Mapped(%) |
| --- | --- | --- | --- | --- | --- | --- |
| SR_1 | 47489996 (99.80%) | 39681213 (83.65%) | 3464 (0.01%) | 52820 (0.11%) | 11.28% | 94.92% |
| SR_2 | 36581000 (99.77%) | 30695949 (84.04%) | 2550 (0.01%) | 46530 (0.13%) | 11.42% | 95.46% |
| SR_3 | 39591732 (99.76%) | 32938661 (83.41%) | 3234 (0.01%) | 60496 (0.15%) | 12.01% | 95.42% |
| SS_1 | 42769572 (99.41%) | 36747547 (86.09%) | 4078 (0.01%) | 147480 (0.34%) | 8.70% | 94.80% |
| SS_2 | 41144790 (99.67%) | 35911338 (87.41%) | 1708 (0.00%) | 73558 (0.18%) | 8.60% | 96.01% |
| SS_3 | 38325696 (99.83%) | 33130647 (86.61%) | 2672 (0.01%) | 38890 (0.10%) | 10.03% | 96.64% |
| Total | 245902786 |  |  |  |  |  |

Table S6. Significant differentially expressed genes (DEGs) between the SS and SR groups.

Due to the large size of the table, please refer to the accompanying Excel file (Supplementary Table S6).

Table S7 Overlapping genes between DAR‑related genes and differentially expressed genes (DEGs) between the SS and SR groups.

Due to the large size of the table, please refer to the accompanying Excel file (Supplementary Table S7).


Table S8. TF_motif in the core promoter region

| motif_ID | motif_alt_ID | consensus | adj_p-value | E-value |
| --- | --- | --- | --- | --- |
| MA0139.2 | CTCF | RCCASYAGRKGGCRS | 1.30×10^-77^ | 1.14×10^-74^ |
| MA1930.2 | CTCF | CTGCAGTKCCNVCHNNYRGCCASYAGRKGGCRS | 2.65×10^-36^ | 2.33×10^-33^ |
| MA1929.2 | CTCF | CTGCAGTKCCNNNNNYNRCCASYAGRKGGCR | 6.52×10^-31^ | 5.73×10^-28^ |
| MA0098.4 | ETS1 | ACCGGAART | 3.49×10^-15^ | 3.06×10^-12^ |
| MA1935.2 | ERF::FOXI1 | AAACMGGAAR | 3.14×10^-13^ | 2.76×10^-10^ |
| MA1952.2 | FOXJ2::ELF1 | AAACMGGAAGT | 1.15×10^-12^ | 1.01×10^-9^ |
| MA0060.4 | NFYA | CCAATCAG | 1.15×10^-12^ | 1.01×10^-9^ |
| MA1942.2 | ETV2::FOXI1 | AAACAGGAAGY | 2.48×10^-12^ | 2.18×10^-9^ |
| MA1644.2 | NFYC | CCAATCA | 5.72×10^-12^ | 5.03×10^-9^ |
| MA0475.3 | FLI1 | ACCGGAART | 1.28×10^-11^ | 1.12×10^-8^ |
| MA0474.4 | Erg | ACAGGAAGTG | 3.17×10^-11^ | 2.79×10^-8^ |
| MA0761.3 | ETV1 | ACAGGAAGT | 5.75×10^-11^ | 5.05×10^-8^ |
| MA0502.3 | NFYB | YCATTGGCC | 9.95×10^-11^ | 8.74×10^-8^ |
| MA2332.1 | ZNF175 | ACAGGAAGT | 1.07×10^-10^ | 9.45×10^-8^ |
| MA1484.2 | ETS2 | ACCGGAAGY | 4.07×10^-10^ | 3.58×10^-7^ |
| MA0697.3 | Zic3 | CAGCAGG | 4.48×10^-10^ | 3.94×10^-7^ |
| MA1483.3 | ELF2 | AMCCGGAAGT | 4.51×10^-10^ | 3.97×10^-7^ |
| MA1508.2 | IKZF1 | AACAGGAA | 5.00×10^-10^ | 4.40×10^-7^ |
| MA2329.1 | ZBTB11 | CACTTCCGG | 6.05×10^-10^ | 5.32×10^-7^ |
| MA0473.4 | ELF1 | CAGGAAGTG | 6.73×10^-10^ | 5.92×10^-7^ |
| MA0598.4 | EHF | CACTTCCTG | 1.40×10^-9^ | 1.23×10^-6^ |
| MA0640.3 | ELF3 | CACTTCCTG | 2.00×10^-9^ | 1.76×10^-6^ |
| MA0750.3 | ZBTB7A | CCGGAAGTG | 2.13×10^-9^ | 1.87×10^-6^ |
| MA1992.2 | Ikzf3 | CAGGAAGTG | 2.43×10^-9^ | 2.13×10^-6^ |
| MA0062.4 | GABPA | CACTTCCTGT | 3.85×10^-9^ | 3.38×10^-6^ |
| MA0764.4 | ETV4 | ACCGGAAGT | 4.54×10^-9^ | 3.99×10^-6^ |
| MA1950.2 | FLI1::FOXI1 | TAAACAGGAAR | 5.65×10^-9^ | 4.97×10^-6^ |
| MA0516.3 | SP2 | GGGGCGGGG | 1.01×10^-8^ | 8.84×10^-6^ |
| MA0076.3 | ELK4 | CRCTTCCGG | 1.03×10^-8^ | 9.08×10^-6^ |
| MA1564.2 | SP9 | CCACGCCCMC | 4.39×10^-8^ | 3.86×10^-5^ |
| MA1102.3 | CTCFL | CAGGGGGC | 4.60×10^-8^ | 4.04×10^-5^ |
| MA0760.2 | ERF | ACCGGAAGT | 6.30×10^-8^ | 5.54×10^-5^ |
| MA0136.4 | Elf5 | AAGGAAGT | 6.95×10^-8^ | 6.11×10^-5^ |
| MA1954.2 | FOXO1::ELK1 | RWMAACAGGAAGT | 6.97×10^-8^ | 6.13×10^-5^ |
| MA0080.7 | Spi1 | AAAGAGGAAGTGG | 1.00×10^-7^ | 8.82×10^-5^ |
| MA1593.2 | ZNF317 | ACAGCAGA | 2.55×10^-7^ | 2.24×10^-4^ |
| MA0645.2 | ETV6 | SCGGAAGTR | 3.82×10^-7^ | 3.36×10^-4^ |
| MA1961.2 | PATZ1 | SGGGGMGGGGS | 5.57×10^-7^ | 4.89×10^-4^ |
| MA2326.1 | IKZF2 | AGGAAG | 8.96×10^-7^ | 7.87×10^-4^ |
| MA1946.2 | ETV5::FOXI1 | GTAAACAGGAWG | 8.97×10^-7^ | 7.89×10^-4^ |
| MA0471.3 | E2F6 | GGCGGGAA | 9.35×10^-7^ | 8.22×10^-4^ |
| MA0762.2 | ETV2 | ACCGGAAAT | 1.05×10^-6^ | 9.24×10^-4^ |
| MA1627.2 | Wt1 | CCTCCCCCAC | 2.38×10^-6^ | 2.09×10^-3^ |
| MA1708.2 | ETV7 | SCGGAAGTR | 2.47×10^-6^ | 2.17×10^-3^ |
| MA1638.2 | HAND2 | CAGATG | 4.04×10^-6^ | 3.55×10^-3^ |
| MA0687.2 | SPIC | AAAAGVGGAAGTA | 4.12×10^-6^ | 3.62×10^-3^ |
| MA1475.2 | CREB3L4 | RTGACGTCA | 4.39×10^-6^ | 3.86×10^-3^ |
| MA0641.1 | ELF4 | AACCCGGAAGTR | 4.92×10^-6^ | 4.32×10^-3^ |
| MA1936.2 | ERF::FOXO1 | RTMAACAGGAAR | 7.20×10^-6^ | 6.33×10^-3^ |
| MA1628.2 | Zic1::Zic2 | CAGCAGG | 7.68×10^-6^ | 6.75×10^-3^ |
| MA1953.2 | FOXO1::ELF1 | RTMAACAGGAAGT | 8.40×10^-6^ | 7.38×10^-3^ |
| MA0028.3 | ELK1 | ACCGGAAGT | 1.30×10^-5^ | 1.14×10^-2^ |
| MA1512.2 | KLF11 | CCACGCCCMC | 1.62×10^-5^ | 1.42×10^-2^ |
| MA1127.1 | FOSB::JUN | GATGACGTCAT | 1.74×10^-5^ | 1.53×10^-2^ |
| MA1145.2 | FOSL2::JUND | RTGACGTCAY | 2.44×10^-5^ | 2.15×10^-2^ |
| MA0081.3 | SPIB | TCACTTCCTCTTT | 2.91×10^-5^ | 2.56×10^-2^ |
| MA1653.2 | ZNF148 | CCCCTCCCCC | 3.17×10^-5^ | 2.78×10^-2^ |
| MA0033.2 | FOXL1 | RTAAACA | 3.79×10^-5^ | 3.33×10^-2^ |
| MA1951.2 | FOS | GATGACGTCATCR | 3.99×10^-5^ | 3.51×10^-2^ |
| MA1139.2 | FOSL2::JUNB | ATGACGTCAT | 5.05×10^-5^ | 4.44×10^-2^ |
| MA0050.4 | Irf1 | TGAAACTGAAA | 5.37×10^-5^ | 4.72×10^-2^ |

Table S9. The top 15 genes annotated with TFs in core promoter regions and genes shared among DEGs

| motif_id | motif_alt_id | p-value | q-value | anno_GeneId | Symbol |
| --- | --- | --- | --- | --- | --- |
| MA0687.2 | SPIC | 4.47×10^-8^ | 0.0358 | ncbi_119888677 | btk |
| MA0080.7 | Spi1 | 5.96×10^-8^ | 0.0177 | ncbi_119916174 | tmem106a |
| MA0687.2 | SPIC | 8.94×10^-8^ | 0.0358 | ncbi_119914142 | galt |
| MA1954.2 | FOXO1::ELK1 | 8.94×10^-8^ | 0.0337 | ncbi_119914142 | galt |
| MA1953.2 | FOXO1::ELF1 | 1.19×10^-7^ | 0.0388 | ncbi_119914142 | galt |
| MA0080.7 | Spi1 | 1.64×10^-7^ | 0.0231 | ncbi_119883055 | nfil3-2 |
| MA1952.2 | FOXJ2::ELF1 | 2.38×10^-7^ | 0.0251 | ncbi_119914142 | galt |
| MA1942.2 | ETV2::FOXI1 | 2.38×10^-7^ | 0.0263 | ncbi_119914142 | galt |
| MA0080.7 | Spi1 | 6.11×10^-7^ | 0.0311 | ncbi_119905688 | bpifcl |
| MA0080.7 | Spi1 | 6.41×10^-7^ | 0.0316 | ncbi_119900956 | chaf1a |
| MA1952.2 | FOXJ2::ELF1 | 7.15×10^-7^ | 0.0473 | ncbi_119889430 | slc25a51b |
| MA1952.2 | FOXJ2::ELF1 | 7.15×10^-7^ | 0.0473 | ncbi_119902041 | irf4a |
| MA0080.7 | Spi1 | 8.49×10^-7^ | 0.0340 | ncbi_119892084 | cmklr1 |
| MA1952.2 | FOXJ2::ELF1 | 9.54×10^-7^ | 0.0495 | ncbi_119891876 | slc2a8 |
| MA0080.7 | Spi1 | 9.83×10^-7^ | 0.0355 | ncbi_119910271 | slc4a11 |
